# Fasting Drives Nrf2-Related Antioxidant Response in Skeletal Muscle

**DOI:** 10.3390/ijms21207780

**Published:** 2020-10-21

**Authors:** Daniele Lettieri-Barbato, Giuseppina Minopoli, Rocco Caggiano, Rossella Izzo, Mariarosaria Santillo, Katia Aquilano, Raffaella Faraonio

**Affiliations:** 1 Department of Biology, University of Rome Tor Vergata, via della Ricerca Scientifica, 00133 Rome, Italy; daniele.lettieri.barbato@uniroma2.it (D.L.-B.); katia.aquilano@uniroma2.it (K.A.); 2 IRCCS Fondazione Santa Lucia, via del Fosso di Fiorano, 00143 Rome, Italy; 3 Department of Molecular Medicine and Medical Biotechnology, University of Naples “Federico II”, via Pansini, 80131 Naples, Italy; giuseppina.minopoli@unina.it (G.M.); rocco.caggiano@unina.it (R.C.); rossellaizzo95@gmail.com (R.I.); 4 Department of Clinical Medicine and Surgery, Physiology Nutrition Unit, Federico II University of Naples, 80131 Naples, Italy; mariarosaria.santillo@unina.it

**Keywords:** nutrient restriction, Nrf2, metabolism, oxidative stress, lipid peroxides

## Abstract

A common metabolic condition for living organisms is starvation/fasting, a state that could play systemic-beneficial roles. Complex adaptive responses are activated during fasting to help the organism to maintain energy homeostasis and avoid nutrient stress. Metabolic rearrangements during fasting cause mild oxidative stress in skeletal muscle. The nuclear factor erythroid 2-related factor 2 (Nrf2) controls adaptive responses and remains the major regulator of quenching mechanisms underlying different types of stress. Here, we demonstrate a positive role of fasting as a protective mechanism against oxidative stress in skeletal muscle. In particular, by using in vivo and in vitro models of fasting, we found that typical Nrf2-dependent genes, including those controlling iron (e.g., *Ho-1*) and glutathione (GSH) metabolism (e.g., *Gcl*, *Gsr*) are induced along with increased levels of the glutathione peroxidase 4 (Gpx4), a GSH-dependent antioxidant enzyme. These events are associated with a significant reduction in malondialdehyde, a well-known by-product of lipid peroxidation. Our results suggest that fasting could be a valuable approach to boost the adaptive anti-oxidant responses in skeletal muscle.

## 1. Introduction

Metabolic adaptation responses are essential to preserve energy homeostasis for basic functions in the organism during fasting. Fasting has a wide range of beneficial effects because it induces protective stress pathways that prevent and/or recover tissue damages; on the contrary, non-physiological, prolonged fasting could be involved in the cell death response governing numerous pathological processes [1,2,3,4,5,6,7,8,9]. Fasting has the potential to counter chronic metabolic disorders and improve health and lifespan; by contrast, misguided responses are potentially devastating and can also contribute to diverse types of muscular dysfunctions, like myotonic dystrophy type 1 [10]. The skeletal muscle activates selective adaptive programs to low-nutrient availability that are mainly characterized by a rapid decrease in glucose oxidation associated with shifts towards lipid catabolic pathways [11,12]. To cope with exhausted reservoirs, skeletal muscle also reduces protein synthesis and activates selective forms of autophagy [13,14,15].

Molecular mechanisms controlling nutrient-induced metabolic reprogramming involve an intricate system of inducers and effectors that primarily boost adaptive metabolic responses and then increase stress resistance to ensure tissue homeostasis and eventually prevent cells from death commitment [2,7,16,17,18,19]. During fasting, bioenergetic intermediate sensors (NAD^+^, AMP, CoA) initiate cellular stress-sensing pathways that further activate energy restriction responses, mostly mediated by PGC-1α and Nrf2 [18,20,21,22,23,24,25]. PGC-1α induces tissue-specific changes in lipid metabolism, tricarboxylic acid cycle and mitochondrial function [26,27,28,29]. Activation of the Nrf2 pathway occurs under the influence of a wide variety of intrinsic or extrinsic stresses that include byproducts of metabolic processes (e.g., ROS), exposure to chemical/environmental agents and conditions of nutrient disturbances (e.g., fasting, overfeeding) [25,29,30,31,32,33,34]. In the field of energy metabolic homeostasis, the Nrf2-driven signaling has recently garnered increasing attention. ROS amounts are indeed modulated by the types of dietary nutrients and increased levels are linked to energy metabolism disturbances. However, Nrf2 signaling could contribute to mounting appropriate responses to nutrient-induced perturbation by influencing various aspects of metabolism [25,29,30,31]. However, the role of Nrf2 in skeletal muscle under nutrient stress conditions remains under-investigated.

In recent years, Nrf2 antioxidant activity has been implicated in protection against ferroptosis, a recently described form of cell death caused by iron-dependent lipid peroxidation [34,35,36,37,38,39,40]. The free form of iron is indeed responsible for the Fenton reaction involving hydroxyl radical production that could propagate and trigger the accumulation of reactive lipid peroxides, the general hallmarks of ferroptosis [40]. Biochemical events with pivotal roles in ferroptosis also involve a severe decrease in the non-enzymatic thiol antioxidant molecule glutathione (GSH) and/or failure in the repair systems that prevent lipid peroxidation. These include glutathione peroxidase 4 (Gpx4) that needs GSH as a reducing cofactor [38,41,42,43]. A broad range of Nrf2-dependent genes can influence the susceptibility to ferroptosis. These genes include those involved in GSH biosynthesis and recycling, in heme and iron homeostasis, and in the NAD(P)H-producing pathway among many others [28,29,34,35,39,41,42,43,44].

Skeletal muscle, together with the liver, contains the highest content of iron in the body. Therefore, it is among the tissues with higher risk to undergo iron-mediated oxidative imbalance. Metabolic adaptation to fasting causes mild oxidative stress and increases long-chain fatty acid metabolism. Thus, it could be inferred that the concomitant presence of fasting-induced oxidative stress and iron can enhance the production of toxic lipids. As a transcription factor, Nrf2 allows appropriate gene expression changes, avoiding the deleterious effects of reactive oxygen species (ROS) and possible functional recovery. Exploration of the Nrf2-mediated control of oxidative stress during fasting in skeletal muscle is lacking. Since fasting signaling could lead to mild oxidative stress, in this paper we explored Nrf2 signaling in skeletal muscle and gave evidence that metabolic reprograming during fasting occurs in parallel with a protective response, resulting in the boosting of antioxidant defense and protection against lipid peroxidation.

## 2. Results

### 2.1. Fasting Modulates the Expression of Genes Involved in Skeletal Muscle Metabolic Adaptations

**Figure 1:**
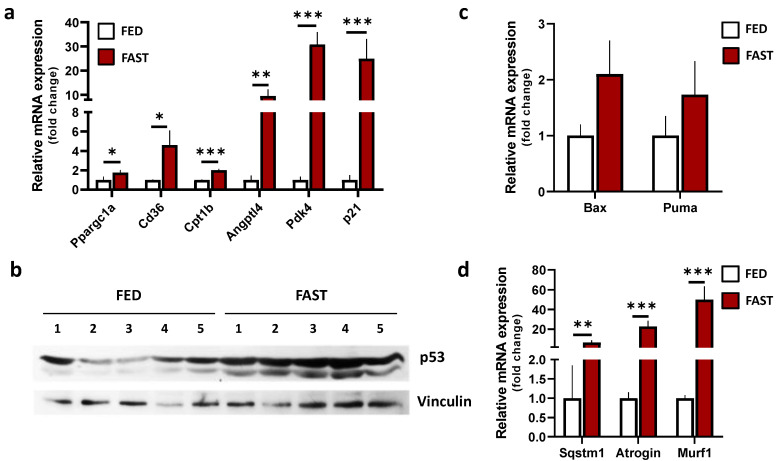
Expression of genes involved in metabolic rearrangements of skeletal muscle during fasting. (**a**) Single gene expression analysis in gastrocnemius of *ad libitum*-fed or 24 h-fasted mice was performed by RT-qPCR. Student *t*-test * *p* < 0.05; ** *p* < 0.01; *** *p* < 0.001 (*n* = 5 mice/group). (**b**) P53 protein levels in gastrocnemius of ad *libitum*-fed or 24 h-fasted mice were analyzed by Western blotting. Vinculin was used as loading control. (**c**,**d**) Single gene expression analysis in gastrocnemius of *ad libitum*-fed or 24 h fasted mice was performed by RT-qPCR. Student *t*-test ** *p* < 0.01; *** *p* < 0.001 (*n* = 5 mice/group). FED: *ad libitum*-fed; FAST: 24 h fasting.

It has been documented that response to fasting includes a tissue-specific transcriptional reprogramming to fine-tune metabolic pathways [45]. To confirm the occurrence of metabolic changes in skeletal muscle following fasting, we collected the total RNAs of skeletal muscle samples from mice after 24 h of food deprivation. The achievement of fasting-induced metabolic remodeling was confirmed by the increased mRNA levels of the master regulator of cell metabolism PGC-1α and its down-stream genes *Cd36* and Carnitine Palmitoyltransferase 1B (*Cpt1b*) required for lipid uptake and degradation, respectively (Figure 1a). We also found elevated mRNA levels of two PPAR-target genes, i.e., angiopoietin-like 4 (*Angptl4*) and pyruvate dehydrogenase lipoamine kinase 4 (*Pdk4*).

p21^CIP1/WAF1^ is another well-characterized fasting-induced gene that participates in physiological adaptation to energy/nutrient stress in skeletal muscle [46]. mRNAs levels of *p21* exhibited a strong increase in fasted mice (Figure 1a), as previously reported [47]. Notably, p53 protein levels were up-regulated as well (Figure 1b), according to the notion that this is a genuine inducer of p21 expression and assists cell-adaptive metabolic responses [48,49]. The mRNAs of canonical p53 down-stream apoptotic genes, such as *Bax* and *Puma*, displayed no statistically significant differences between the two groups, excluding a pro-apoptotic function of p53 in this context (Figure 1c). We also analyzed whether pathways contributing to satisfying energy requirements during fasting, such as autophagy and/or proteasome-dependent protein degradation, could be operative. The analysis of the mRNA level of *Sqstm1/p62*, which is typically related to autophagy, revealed that it was up-regulated (Figure 1d). Moreover, the mRNA expression of the E3 ubiquitin ligases *MuRF1* and *Atrogin-1* (Figure 1d), pertaining to the proteasome-mediated degradation system, were increased, indicating a multimodal way to increase amino acid disposal following fasting. In line with the published literature, the above results indicate that, in skeletal muscle upon 24 h of fasting, a metabolic remodeling supported by specific transcriptional changes was occurring.

### 2.2. Fasting Induces Nrf2-Related Stress Response Pathways in Skeletal Muscle

**Figure 2:**
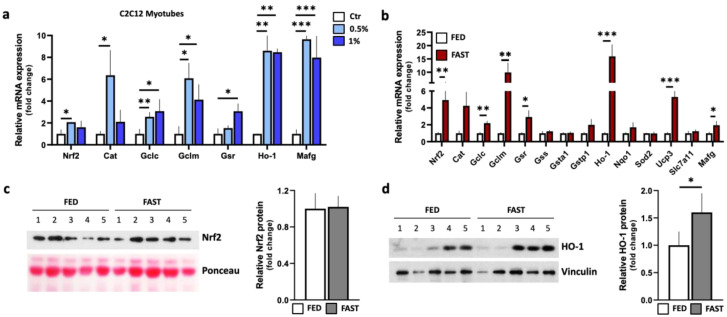
Expression of Nrf2-dependent genes in skeletal muscle during fasting. (**a**) Single gene expression analysis in 24 h serum-starved (0.5% or 1%) C2C12 myotubes or controls (ctr) was performed by RT-qPCR. Student *t*-test * *p* < 0.05; ** *p* < 0.01; *** *p* < 0.001 (*n* = 6 independent experiments). (**b**) Single gene expression analysis in gastrocnemius of *ad libitum*-fed or 24 h-fasted mice was performed by RT-qPCR. Student *t*-test * *p* < 0.05; ** *p* < 0.01; *** *p* < 0.001 (*n* = 5 mice/group). (**c**) Nrf2 protein levels (left panel) in gastrocnemius of *ad libitum*-fed or 24 h-fasted (fast) mice were analyzed by Western blotting. Ponceau staining was used as loading control. Densitometric analysis (right panel) was performed and results expressed as relative Nrf2 protein (Nrf2 to Ponceau ratio) (*n* = 5 mice/group). (**d**) HO-1 protein levels (left panel) in gastrocnemius of *ad libitum*-fed or 24 h-fasted mice were analyzed by Western blotting. Vinculin was used as loading control. Densitometric analysis (right panel) was performed and results expressed as relative HO-1 protein (HO-1 to Vinculin ratio). Student *t*-test * *p* < 0.05; ** *p* < 0.01; *** *p* < 0.001 (*n* = 5 mice/group). FED: *ad libitum*-fed; FAST: 24 h fasting.

It is now widely ascertained that metabolic adaptation during fasting enhances mitochondrial function and oxidative phosphorylation rate in skeletal muscle and, as a result, ROS production is increased [29]. Hence, it appeared plausible that Nrf2 signaling could be activated in association with the observed metabolic reprogramming. We hence moved towards preliminarily analyzing the canonical Nrf2-mediated stress response pathway in a skeletal muscle cellular model. In particular, we used C2C12 myoblasts that can differentiate into myotubes. C2C12 myoblasts were cultured for 5 days in a low-serum (2%) differentiation medium and then maintained for 24 h in further reduced glucose (5.5 mM) and serum concentrations (0.5% or 1% serum), to mimic fasting conditions (Supplementary Figure S1). As shown in Figure 2a, mRNAs levels of *Nrf2* and its well-known downstream genes controlling the response to oxidative stress, i.e., catalase (*Cat*), γ-GCL subunits (*Gclc*, *Gclm*), glutathione reductase (*Gsr*) and heme oxygenase-1 (*Ho-1*) were upregulated. 

Next, we moved at analyzing the Nrf2 stress response in vivo by evaluating the expression of a number of canonical Nrf2-dependent genes as well, like *Cat*, *Gclc* and *Gclm*, *Gsr*, Glutathione synthetase (*Gss*), glutathione S-transferases (*Gsta1, Gstp1*), *Ho-1*, NAD(P)H quinone-oxidoreductase 1 (*Nqo1*), manganese superoxide dismutase 2 (*Sod2*), uncoupling protein 3 (*Ucp3*) and the *x-CT* component (cystine/glutamate transporter subunit) of the cystine/glutamate exchange transport system (*Slc7a11/x-CT*). RT-qPCR analyses showed that, along with increase of *Nrf2* mRNA levels, an up-regulation of *Cat*, *Gclc*, *Gclm*, *Gsr*, *Ho-1* and *Ucp3* gene expression was achieved during fasting in skeletal muscle (Figure 2b), even though the total protein content of Nrf2 remained unchanged (Figure 2c). We also looked at mRNA levels of *Mafg* both in C2C12 and skeletal muscle, with this factor being an essential heterodimeric partner for DNA binding and activity of Nrf2 [50]. As demonstrated by RT-qPCR, we found that the mRNA levels of *Mafg* were upregulated (Figure 2a,b), indicating its contribution to fasting-induced stress responses. According to RT-qPCR analyses, Western blotting revealed that the levels of Ho-1 protein are also increased further supporting Nrf2-mediated activity on *Ho-1* transcription regulation (Figure 2d).

### 2.3. Fasting Reduces Lipid Peroxidation in Skeletal Muscle

**Figure 3:**
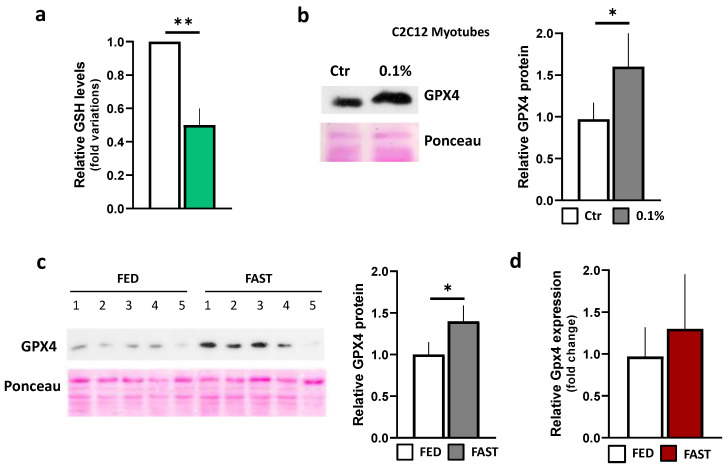
Analysis of GSH/GPX4 system in skeletal muscle during fasting. (**a**) Glutathione (GSH) level was measured in gastrocnemius of *ad libitum*-fed or 24 h-fasted mice by HPLC. Student *t*-test ** *p* < 0.01 (*n* = 5 mice/group). (**b**,**c**) GPX4 protein levels in 24 h serum-starved (1%) C2C12 myotubes or controls (Ctr) (**b**), and in gastrocnemius of *ad libitum*-fed or 24 h-fasted mice (**c**) were analyzed by Western blotting. Ponceau staining was used as loading control. Densitometric analyses (right panels) were performed and results expressed as relative Gpx4 protein (Gpx4 to Ponceau ratio). Student *t*-test * *p* < 0.05 (**b**) *n* = 6 independent experiments; (**c**) *n* = 5 mice/group. (**d**) Single gene expression analysis in gastrocnemius of *ad libitum*-fed or 24 h-fasted mice was performed by RT-qPCR (*n* = 5 mice/group). FED: *ad libitum-fed*; FAST: 24 h fasting.

Overall, these results suggested that Nrf2 activation, in this context, could be a relevant means to fine-tune the intracellular pathways related to ROS detoxification and GSH metabolism. Hence, we measured GSH concentrations in skeletal muscle after fasting and found that they were significantly reduced compared to fed tissues (Figure 3a). Intracellular levels of GSH are usually reduced as consequence of its direct scavenging activity against ROS and indirect buffering effects against lipid peroxides, being a cofactor of Gpx4 activity [38,43]. We hence analyzed Gpx4 protein through Western blot. As reported in the Figure 3b,c, we detected a significant increase in Gpx4 protein levels both in starved C2C12 cells and in the skeletal muscle of fasted mice; however, the Gpx4 mRNA levels in skeletal muscle were not significantly up-regulated (Figure 3d), indicating that Gpx4 protein stabilization is a possible mechanism engaged to limit lipid damage during fasting, arguing that the increase in Gpx4 protein may depend on its increased protein stability.

**Figure 4:**
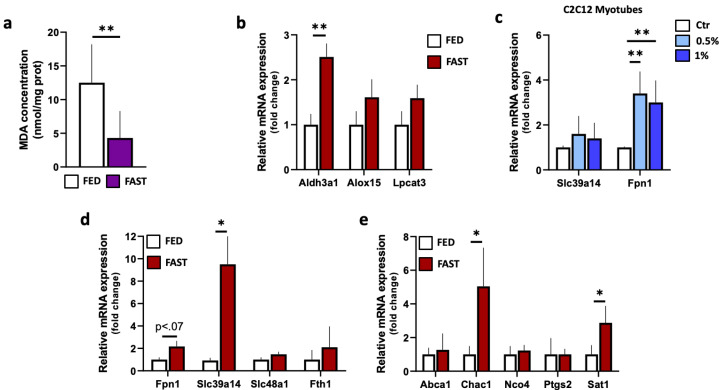
Analysis of lipid peroxidation and genes controlling iron metabolism in skeletal muscle during fasting. (**a**) Malondialdehyde (MDA) levels were measured in gastrocnemius of *ad libitum*-fed or 24 h-fasted mice by a colorimetric assay kit. (**b**) Single-gene expression analysis in gastrocnemius of *ad libitum*-fed or 24 h-fasted mice was performed by RT-qPCR. Student *t*-test ** *p* < 0.01 (*n* = 5 mice/group). (**c**) Single gene expression analysis in 24 h serum-starved (1% or 0.5%) C2C12 myotubes or controls (ctr) was performed by RT-qPCR. Student *t*-test ** *p* < 0.01 (*n* = 6 independent experiments). (**d**,**e**) Single gene expression analysis in gastrocnemius of *ad libitum*-fed or 24 h-fasted mice was performed by RT-qPCR. Student *t*-test * *p* < 0.05 (*n* = 5 mice/group). FED: *ad libitum*-fed; FAST: 24 h fasting.

Oxidative stress can induce lipid peroxidation. Among the aldehydes derived by lipid peroxidation, malonaldehyde (MDA) is considered a valid marker to monitor the peroxidation of polyunsaturated fatty acids. We investigated MDA levels by using a colorimetric method. In line with enhanced Gpx4 and decreased GSH levels, upon fasting we found a significant reduction in MDA levels (Figure 4a), pointing to a protective function of fasting against the peroxidation of lipids. This assumption is also supported by the increased mRNA amounts of *Aldh3a1* (Figure 4b), an enzyme that specifically eliminates highly reactive aldehydes generated during lipid peroxidation at membrane sites. Of note, Aldh3a1 is tightly associated with Nrf2, being a direct target of Nrf2 signaling [50,51]. Notably, the expression of genes related to the oxidation and generation of PUFAs such as *Alox15* [52] and *Lpcat3* [36], respectively, remained unchanged (Figure 4b).

One of the best-known examples of Nrf2-regulated gene is *Ho-1* that, once induced via the Nfr2/Mafg activity, cleaves heme into biliverdin, which is in turn rapidly converted to bilirubin, carbon monoxide (CO) and ferrous iron (Fe^2+^) [53]. Since we have found a strong enrichment of *Ho-1* mRNA and protein in the skeletal muscle of fasted mice (Figure 2b,d), we hypothesized that skeletal muscle responses to nutrient stress could involve the induction of iron-scavenging mechanisms in order to avoid free iron overload and thus prevent possible lipid peroxidation. To test this, we measured the mRNA levels of the divalent metal transporter *Slc39a14/Zip14* and the iron efflux pump ferroprotin (*Fpn1*). As shown in Figure 4c,d, the measurement of *Fpn1* mRNAs revealed a significant increase in starved C2C12 cells and an up-regulation trend upon fasting in vivo, indicating that fasting could increase iron efflux. Slc39a14, besides mediating the transport of manganese and cadmium, also exports iron [54]. In the skeletal muscle of fasted mice, we evidenced a significant increase in *Slc39a14* mRNA, arguing that iron homeostasis could be preferentially maintained by this system in vivo. In line with this idea, the heme transporter *Slc48a1* and ferritin heavy chain 1 (*Fth1*) mRNA levels remained unaltered in skeletal muscle of fasted mice (Figure 4d).

Finally, through RT-qPCR, we measured the expression of a cluster of genes that directly or indirectly have been correlated to lipotoxicity/ferroptosis, including *Abca1*, *Chca1*, *Nco4*, *Ptgs2*/*Cox2* and *Sat1.* Among the mRNAs tested, only *Chac1* and *Sat1* were augmented; whereas *Abca1*, *Nco4* and *Ptgs2* (a putative marker used to prove ferroptosis in vivo) were not changed and displayed no statistically significant differences between the groups (Figure 4e).

The above results indicate that upon fasting, skeletal muscle activates antioxidant Nrf2-mediated pathway and, in parallel, increases Gpx4 expression, thus preventing lipid peroxidation.

## 3. Discussion

Skeletal muscle is considered an important regulator of the metabolism in the whole body [55,56]. Fasting without loss of essential nutrients has the potential to improve health, and research behind it has revealed benefits on health and lifespan by boosting endogenous stress defenses, lowering cardiovascular risk and preventing diabetes [1,2,3,4,6,7]. Therefore, deep exploration of the protective functions of fasting at molecular level could contribute to advantageously managing metabolic pathway to achieve physiological protection from oxidative stress and potentially match organ regenerative activities. Lipid toxicity and consequent ferroptosis have been implicated in the pathogenesis of different disorders including neurodegenerative diseases, acute renal failure, cardiomyopathies and cancer [56,57,58,59]. We suppose that fasting could be a possible route compared to oxidative stress-mediated responses that could, in turn, protect against lipid peroxidation in skeletal muscle, an iron-rich tissue, where redox balance can be perturbed for numerous physiological (e.g., contraction) as well as pathological conditions [60,61,62]. To gain deeper insight, we have investigated many molecular components associated with these mechanisms in vivo. Considering the role of Nrf2 in the transcriptional control of intermediary metabolism and protective antioxidant genes [25,39,63,64], we assessed whether Nrf2-related cell response is induced under fasting conditions. Among the genes analyzed, we found the induction of some Nrf2-target genes, including those involved in GSH biosynthesis and recycling, as well as in heme metabolism, like *Ho-1*, the master regulator of heme level, and others implicated in mitochondrial performance and ROS scavenging, like *Ucp3* and *Cat*, respectively. GSH is a necessary cofactor for protective antioxidant reactions, such as those involving Gpx4, whose activity, if extremely reduced, activates the ferroptotic program [38,43]. Indeed, by exploiting the reducing equivalents of GSH, Gpx4 counteracts the random oxidation of membrane phospholipids by ROS [38,43,65]. Upon fasting, we found a significant increase in Gpx4 protein content in concomitance with the reduction in MDA levels, suggesting that Gpx4 is actively involved in lipid peroxide scavenging in skeletal muscle. In line with this concept, in parallel to Gpx4 up-regulation and MDA level reduction, we observed a mild but significant decrease in intracellular GSH concentration. However, our data suggest that GSH homeostasis is still maintained, since the Nrf2-dependent genes involved in its biosynthesis (e.g., *Gclc*, *Gclm*) and recycling (i.e., *Gsr*) are up-regulated in response to fasting. Thus, it is likely that antioxidant Nrf2-mediated response in skeletal muscle could serve to control fasting-induced redox imbalance, thus avoiding possible lipid peroxidation linked to variation in lipid catabolism, as demonstrated by reduced MDA levels and up-regulation of Gpx4 enzyme and *Aldh3a1* mRNA.

We cannot exclude that the decrease in GSH levels could at least, in part, also derives from its direct ROS scavenging activity or the increased Chac1 expression, a protein factor that specifically breaks GSH upon endoplasmic reticulum (ER) stress conditions. If ER stress is operative under fasting remains however to be elucidated, as it was beyond the scope of our work. As a second Nrf2-related protective mechanism induced by fasting, we found the up-regulation of Ho-1. The function of Ho-1 in cell death, like ferroptosis is controversial, with studies reporting that it can either promote or inhibit ferroptosis [44,66,67,68]. Indeed, Ho-1, by degrading heme, could increase the intracellular concentrations of labile iron. By contrast, Ho-1 produces biliverdin, which is considered a potent antioxidant that could prevent adverse oxidative cascades. We found that in skeletal muscle, to buffer the possible harmful effects of iron associated with increased Ho-1 levels, iron-scavenging mechanisms are up-regulated that ar based on the zinc metal transporter Slc39a14/Zip14 or on the iron efflux pump Fpn1. Of note, the mRNA expression of the ferritin heavy chain (*Fth*) and the ferritinophagy modulator *Ncoa4* remained unchanged, pointing to a regulated role of iron export during fasting.

In our opinion, fasting-induced mild oxidative stress response primes the skeletal muscle to better withstand oxidative stress, which normally occurs during physiological contraction and/or regular physical activity. Thus, this Nrf2-driven antioxidant mechanism could change the set point of stresses and/or points to adaptive transcriptional program buffering future stressors, leading to beneficial effects on health and lifespan.

## 4. Materials and Methods

### 4.1. Animal Husbandry and Treatments

Mouse experimentation was conducted in accordance with the accepted standard of humane animal care after the approval by relevant local (The University Animal Welfare Committee—OPBA, Tor Vergata University) and national (Ministry of Health, Legislative Decree No. 26/2014; European Directive 2010/63/UE) authorities. C57BL/6J. Adult (3 months-age-old) male mice (purchased from ENVIGO, Italy) were housed one per cage, with a 12-h light/dark cycle, at 23–25 °C. All animals had free access to water and were randomly assigned to the experimental groups. Mice fasted for 24 h (FAST) were compared with age and sex-matched mice fed with control diet (*ad libitum*-fed mice: FED). Animals were sacrificed in a randomized order to minimize experimental bias, and skeletal muscle gastrocnemius was harvested.

### 4.2. Cells and Treatments

C2C12 mouse myoblasts were purchased from ATCC (American Type Culture Collection, Bethesda, MD, USA). The cells were seeded on Falcon dishes at 37 °C with 5% CO_2_ in growth medium (Dulbecco modified Eagle medium, DMEM), supplemented with 10% heat-inactivated fetal bovine serum (Thermo FisherSCIENTIFIC, Italia, Monza, Italy), (100 U/100 g/mL) penicillin-streptomycin (Thermo FisherSCIENTIFIC). For differentiation experiments, growth medium was replaced by differentiation medium (DMEM with 2% horse serum, (Thermo FisherSCIENTIFIC)) when cells reached 90% confluence. At 5 days of differentiation, myotubes were cultured in 1% or 0.5% serum and 5.5 mM glucose, or complete (ctr) medium for further 24 h.

### 4.3. RT-qPCR Gene Expression Analysis

Total RNA from tissues and cells was isolated with TRIzol Reagent (Thermo FisherSCIENTIFIC) following the manufacturer’s instruction. cDNA was synthesized from one µg of RNA using SensiFAST cDNA Synthesis Kit (Bio-Line, Aurogene, Italy). mRNA levels were quantified using SensiFAST SYBR No-ROX (Bio-Line) on the CFX96 real-time system instrument (Bio-Rad, Hercules, CA, USA). All the PCR reactions were performed in triplicate and the protocol was carried out according to the manufacturer’s instructions. The housekeeping *Gapdh* or *c-Abl* genes were used for internal normalization. The sequences of mouse primers used in this study are reported in Supplemental Table S1. Relative fold variations were calculated using the 2^−ΔΔCt^ method.

### 4.4. Immunoblotting Analysis

Tissues (100 mg of gastrocnemius) or cells were lysed in RIPA buffer (50 mM Tris-HCl, pH 8.0, 150 mM NaCl, 12 mM 464 deoxycholic acid, 0.5% Nonidet P-40, and protease and phosphatase inhibitors). After quantification, 40 μg proteins were loaded on SDS-PAGE. The proteins were transferred to PVDF membranes (Immobilon-P, Millipore, Merk Life Science, Milan, Italy) and incubated with the primary antibodies overnight at 4 °C. After washing, the appropriate horseradish peroxidase-conjugated secondary antibodies were used to detect immunoreactive bands with Clarity Western ECL substrate chemiluminescent detection reagent (Bio-Rad).

Normalization of Western blotting with housekeeping Vinculin bands was performed with the ImageJ software [69]. Relative band intensities on the Ponceau-S staining (Sigma-Aldrich, Milan, Italy) of the PVDF membranes were determined by using the Band Analysis tools of ImageLab software version 4.1 (Bio-Rad) prior incubation with primary antibodies.

Antibodies used in this study were: anti-Gpx4 (Proteintech, Manchester, UK); Anti Ho-1 (Santa Cruz Biotechnology, Santa Cruz, CA, USA); anti-Nrf2 (Santa Cruz Biotechnology); anti-p53 (Santa Cruz Biotechnology); anti-Vinculin (Santa Cruz Biotechnology); anti-rabbit IgG HRP conjugate Normal rabbit IgG (Santa Cruz Biotechnology); anti-goat IgG HRP-conjugate (Sigma-Aldrich); anti-rabbit IgG HRP-conjugate (GE Healthcare); anti-mouse IgG HRP-conjugate (GE Healthcare, Sigma-Aldrich).

### 4.5. GSH and MDA Measurements

In the gastrocnemius of mice, the GSH levels were measured by HPLC as previously described [2]. The MDA amounts were measured on 10 mg of tissue by using a commercially available kit for lipid peroxidation (MDA) (Sigma-Aldrich) according to manufacturer’s instruction.

### 4.6. Statistical Analyses

The raw data were analyzed using Student *t*-test and represented as means ±SD. The differences were considered significant at *p* < 0.05. The number of animals and in vitro experiments were reported in the figure legends. Data were analyzed with Excel and GraphPad Prism 8 (La Jolla, CA, USA).

## 5. Conclusions

This study highlights that nutrient deprivation/fasting could be an appropriate strategy to boost antioxidant defenses and protect against lipid peroxidation. The Nrf2 transcription factor coordinates an adaptive program that controls iron homeostasis and helps biosynthesis of glutathione, which is essential for Gpx4 activity. Exposure to fasting could be an opportunity to prevent different disorders, likely related to events that can activate the ferrotoptic process.

## Supplementary Materials

Supplementary Materials can be found at https://www.mdpi.com/1422-0067/21/20/7780/s1. Figure S1: Representative images of C2C12 myotubes subjected to fasting-mimicking culture conditions, Table S1: List of oligos used in this study.
