# Supplementary material for: Fasting Drives Nrf2-Related Antioxidant Response in Skeletal Muscle
Source: Int J Mol Sci. 2020 Oct 21;21(20):7780. doi: 10.3390/ijms21207780 (PMC7589317; doi:10.3390/ijms21207780)
Supplement: Supplementary file 1 [file ijms-21-07780-s001.zip › Table S1 Oligos.docx]

Table S1. List of oligos used in this study

|  | **PRIMERS** | |
| --- | --- | --- |
|  | **Forward** | **Reverse** |
| Abca1 | 5’ - CCGGCTTGGGGAGCTG - 3’ | 5’ - CTGTGACTCTGTGGCTGGTC - 3’ |
| Aldh3a1 | 5’ - CATCTGACCCCTGTCACCTT - 3’ | 5’ - CCCATAGTCATGGGACTGCT - 3’ |
| Alox15 | 5’ - GGTGCTGAAGCGGTCTACTT - 3’ | 5’ - TTGGCCTTGATCCCATCCAG - 3’ |
| Angptl4 | 5’ - AGCTCATTGGCTTGACTCCC - 3’ | 5’ - GAAGTCCACAGAGCCGTTCA - 3 |
| Atrogin | 5’- CAGCAGCCTGAACTACGACG - 3’ | 5’ - GGCAGTCGAGAAGTCCAGTC - 3’ |
| BAX | 5’ - ACAGATCATGAAGACAGGGG - 3’ | 5’ - CAAAGTAGAAGAGGGCAACC - 3’ |
| c-ABL | 5′ -GGTATGAAGGGAGGGTGTACCA-3′ | 5′ - GTGAACTAACTCAGCCAGAGTGTTGA - 3′ |
| Cat | 5’ - CGTCCCTGCTGTCTCACGTT - 3’ | 5’ - ATCTCCTATTGGGTTCCCGC - 3’ |
| Cd36 | 5’ - ATGGGCTGTGATCGGAACTG - 3’ | 5’ - TTTGCCACGTCATCTGGGTTT - 3’ |
| Chac1 | 5’ - GCCCTGTGGATTTTCGGGTA - 3’ | 5’ - CGGTCTTCAAGGAGGGTCAC - 3’ |
| Cpt1b | 5’ - TGTCTACCTCCGAAGCAGGA - 3’ | 5’ - CGGCTTGATCTCTTCACGGT - 3’ |
| Fth1 | 5’ - GCCAGAACTACCACCAGGAC - 3’ | 5’ - GCCAGAACTACCACCAGGAC - 3’ |
| Gapdh | 5’ - AACATCAAATGGGGTGAGGCC - 3’ | 5’ - GTTGTCATGGATGACCTTGGC - 3’ |
| Gclc | 5’ - TGCACATCTACCACGCAGTC - 3’ | 5’ - GGATGGTTGGGGTTTGTCCT - 3’ |
| Gclm | 5’ - AGTTGGAGCAGCTGTATCAGTGG - 3’ | 5’ - TTTAGCAAAGGCAGTCAAATCTGG - 3’ |
| Gpx4 | 5’ - TTACTTAAGCCAGCACTGCTGTG - 3’ | 5’ - CCATGTGCCCGTCGATGT - 3’ |
| Gr | 5’ - CGAAGCTGTTCATAAGTATGGGA - 3’ | 5’ - CCTGCATGTGAATGCCAAACC - 3’ |
| Gss | 5’ - ACAACGAGCGAGTTGGGATG - 3’ | 5’ - CAGAGCACTGGGTACTGGTG - 3’ |
| Gsta1 | 5’ - CAGGTGGCTCCTAGCTGCA - 3’ | 5’ - GGTCTGCGCCAGCTTCA - 3’ |
| Gstp1 | 5’ - TTCTCTCTGCACAGCAGCCA - 3’ | 5’ - AACCACCTCCTCCTTCCAGC - 3’ |
| Ho-1 | 5’ - AGGATTTGTCTGAGGCCTTG - 3’ | 5’- AGGAAGCCATCACCAGCTTA - 3’ |
| Lpcat3 | 5’ - TGCGGCTCATCTTCTCCATC - 3’ | 5’- AATTGAGAGGCCCGTGAAGG - 3’ |
| Mafg | 5’ - TGTGAGTGCCTGCTCACTGT - 3’ | 5’ - GTCAAGCTGGTGCCATTCTC - 3’ |
| Murf1 | 5’ - Acctgctggtggaaaacatc - 3’ | 5’ - cttcgtgttccttgcacatc - 3’ |
| Ncoa4 | 5’ - TGTCCCTTTAACACTGCCGA | 5’ – GAAATGCTTCCTGTGCCTTCTT – 3’ |
| Nqo1 | 5’ - CCCTCAACATCTGGAGCCAT - 3’ | 5’ - GCGTAGTTGAATGATGTCTTCTCTGA - 3’ |
| Nrf2 | 5’ - GGCCCAGCATATCCAGACA - 3’ | 5’ - CCAGGGCAAGCGACTCAT - 3’ |
| p21 | 5’ - CCACAGCGATATCCAGACATTC - 3’ | 5’ - CGAAGAGACAACGGCACACTT - 3’ |
| Pdk4 | 5’ - CTGCCTGACCGCTTAGTGAA - 3’ | 5’ - TGCCTTGAGCCATTGTAGGG - 3’ |
| Ppargc1a | 5’ - CAGTACTGCCACTGCGATCA - 3’ | 5’ - GGCCAGGATGTTGGTTAGCT - 3’ |
| Ptgs2 | 5’ - tgtgaagggaaataaggagctt - 3’ | 5’ - GGGATACACCTCTCCACCAA - 3’ |
| Puma | 5’ - ACAGATCATGAAGACAGGGG - 3’ | 5’ - AGTCCCATGAAGAGATTGTACATGAC -3’ |
| Sat1 | 5’ – GGACCCCTGAAGGACATAGC - 3’ | 5’ – ACTTGCCAATCCATGGGTCAT – 3’ |
| Slc7a11 | 5’ -ACCTCAACTTTATTACTGAAGAAGTAGACAA - 3’ | 5’ - TGTCAGTACGTAGCCCACTGTGA - 3’ |
| Slc40a1 | 5’ - CAGTACTGCCACTGCGATCA - 3’ | 5’ - GGCCAGGATGTTGGTTAGCT - 3’ |
| Slc48a1 | 5’ - GCCGGCTTCTCCATCTTCTT - 3’ | 5’ - TGAGCCAGGTCCTCCAGTAA - 3’ |
| Sod2 | 5’ - GTCGCTTACAGATTGCTGCCT - 3’ | 5’ - AGGTAGTAAGCGTGCTCCCACA - 3’ |
| Sqstm1 | 5’ - AGAATGTGGGGGAGAGTGTG - 3’ | 5’ - TTTCTGGGGTAGTGGGTGTC - 3’ |
| Ucp3 | 5’ - TGCTGAAGATGGTGGCTCAG - 3’ | 5’ - GGCCCTCTTCAGTTGCTCAT - 3’ |
