# Supplementary figures and images for: Fasting Drives Nrf2-Related Antioxidant Response in Skeletal Muscle
Source: Int J Mol Sci. 2020 Oct 21;21(20):7780. doi: 10.3390/ijms21207780 (PMC7589317; doi:10.3390/ijms21207780)

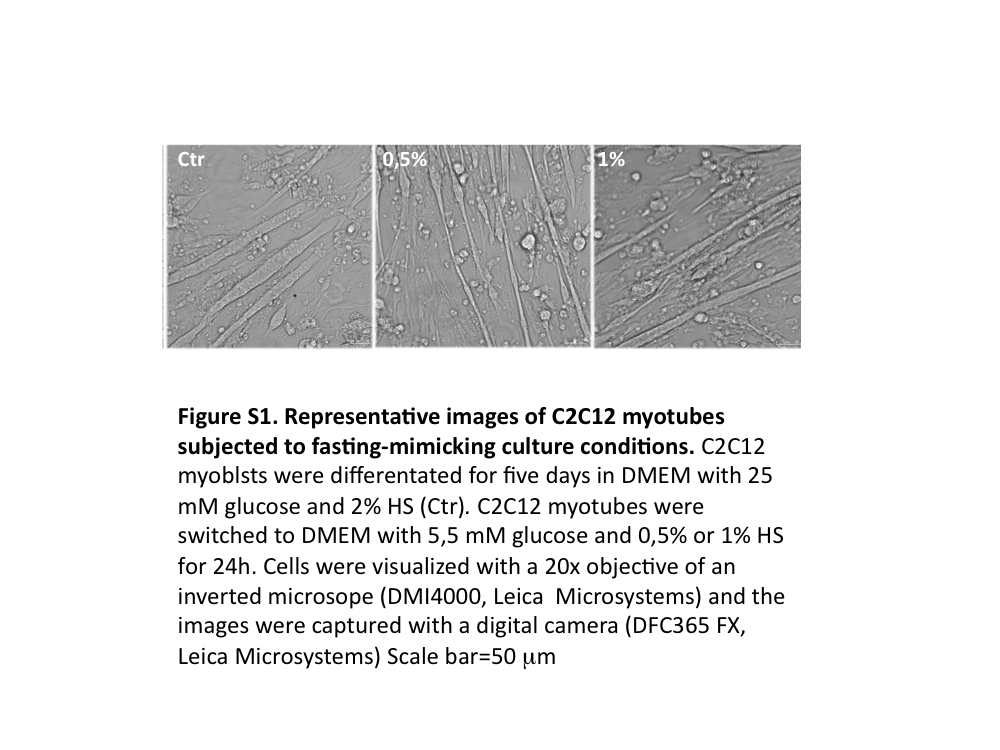

Supplement: Supplementary file 1 [file ijms-21-07780-s001.zip › Figure S1.tif]
